# Ideals for the Future

**Published:** 1916-04-29

**Authors:** 


### THE SCHOOL DOCTOR.

### Ideals for the Future.

When the recruiting returns are published at the conclusion of the war, it is safe to prophesy that the enormous numbers of men rejected as "physically unfit," together with the thousands more who broke down during training, will form a powerful argument for the extension of the school doctor's work. But there are *culs-de-sac* on the path of school hygiene, as of all other progressive movements, and it is essential to make clear the lines on which expansion may most profitably take place. The department of school hygiene on which most work has been concentrated of late years is that of inspection of the children and the recording of defects. Here, if anywhere, there is room for pruning, for with three detailed routine inspections in every child's school life, coupled with the daily observation of increasingly alert and well-instructed teachers, very few defects can escape unnoted. Now it is commonly accepted that the first duty that a doctor has towards any physical ailment is to secure treatment for it with the least possible delay. The school medical service is the only known exception. The defects of school children are recorded on a dozen separate forms and cards, and are made the basis of a vast and valueless mass of statistics, absorbing the energies of hundreds of clerks, and covering acres of paper. Yet, as a conclusion, the Board of Education's annual report for 1914 has to record that "up to the present time *not more than half* the children suffering from severe but remediable defects are cured of them during their school life." About 50 per cent. of the children are no whit the better for the considerable expenditure of public money. Only 26.1 per cent. of all nose and throat cases, and 46.5 per cent of vision defects are adequately dealt with. Reports from quite large towns and counties record year after year that 60 per cent. to 70 per cent. of their defective children are getting no treatment, mostly for the excellent reason that no treatment is to be had. Fifty-one Local Authorities have still made no arrangements for treatment whatever, and there are areas where the best intentioned parents can get nothing done for carious teeth, or otorrhœa, or ringworm.

### A Lack of Patriotism.

These facts demonstrate a grievous lack of patriotism on the part of Local Authorities, and challenge the professional honour of the school doctor. Here, too, is a most obvious field for the profitable expenditure of public funds. The first step is the organisation of pre-existing remedial agencies, such as private practitioners and voluntary hospitals, and where these prove inadequate, the establishment of school clinics; 350 such centres were sanctioned by the Board for 1915, but very many more are required before the ground is covered. Wherever they have been tried it is found that the standard of child care for the whole neighbourhood is raised, and the parents' sense of responsibility increased; nor can any vested interests be violated by the treatment of such defects. The question of the treatment of the individual child is still far from being satisfactorily solved, but there is another department of school hygiene even more neglected. The *prevention* of ill-health by the study of hygienic difficulties peculiar to schools, and the improvement of unfavourable conditions of every description was an important part of the work of the early school doctors, yet all reference to it is omitted from many of the present school medical officers' reports. Little or no time is allowed for original investigations in many districts, suggested improvements in school conditions are not made, or are politely ignored by the committees. School doctors are still found protesting that they cannot secure a decent and reasonable standard of cleanliness in the schools under their care, while much educational and monetary loss is occasioned by the "dirt diseases." Again, child rheumatism costs the community very large sums annually in loss of attendance grant, and the provision of special schools for heart cases, not to mention the expenses of invalidity in later life; yet the little that we could do to prevent it is not done. It is the exception to find proper arrangements in an elementary school for drying outdoor clothes or changing wet shoes. As school doctors have pointed out *ad nauseam*, dark and over-crowded cloakrooms directly favour the spread of pediculi which we are employing an army of nurses to check. Instances could be multiplied indefinitely to show that if it is poor economy to leave known defects untreated, it is still more wasteful to suffer unhygienic conditions which produce such defects.

### The Clinical Psychologist.

The task of perfecting the hygiene of school buildings involves a close co-operation between the doctor and the architect. Physiological research is constantly modifying our knowledge of the health requirements of teacher and child, and there is considerable danger that, unless an architect is kept in close communication with the medical officer, he may be working in conformity with obsolete formulæ. Recent revolutionary discoveries in ventilation and the importance now attached to the physical properties of air rather than to its carbonic-acid content, have made some of our schools out of date before they have been erected.

Many educational problems long awaiting a solution require investigation from the standpoint of psychological medicine and psychiatry. For instance, recent intensive studies into the causes of retardation, particularly those of Dr. Hutt, of Brighton, show that in an enormous percentage of cases physical defects and social conditions are at fault, and teachers have been unjustly blamed for many educational failures for which neither they nor the school were responsible. In this connection an interesting innovation, the clinical psychologist, has made his appearance, chiefly in America. This expert comes equipped with a fearsome jargon and mysterious technique, whereby he claims to diagnose and treat the whole range of mental and moral abnormalities and neuroses of childhood. The services of the medical man are requisitioned (if at all) only for the removal of adenoids or the provision of spectacles. While recognising the great utility of psychology in medicine and education, it is important to avoid the pitfalls of quackery.

It is perhaps only those engaged in school hygiene who can realise the extent to which the modern elementary school has become the centre of "child welfare" work. Hordes of voluntary philanthropic workers, psychologists, physical trainers, lecturers on health and morals, are invading the domain which was once the peaceful preserve of the teacher and the parson. It is becoming an increasingly important function of the school doctor to co-ordinate and guide every influence affecting even remotely the health of the children. He must be prepared to utilise every source of expert knowledge, to stimulate philanthropic devotion to the children's service, and at the same time to protect the little ones from one-sided enthusiasts and faddists.

The school doctor who is content to be a mere docile diagnostician, an automaton for recording the physical state of children with whose health he has no further concern, is indeed an extravagance. The need for national economy and the dignity of the medical profession cry aloud for his abolition. But the school doctor who is able and willing to become a controlling factor in the welfare of our child population is an asset which the nation simply cannot afford to do without.

